# Age-Related Variations in Intestinal Microflora of Free-Range and Caged Hens

**DOI:** 10.3389/fmicb.2017.01310

**Published:** 2017-07-11

**Authors:** Yizhe Cui, Qiuju Wang, Shengjun Liu, Rui Sun, Yaqiang Zhou, Yue Li

**Affiliations:** College of Animal Science and Veterinary Medicine, Heilongjiang Bayi Agricultural University Daqing, China

**Keywords:** hens, feeding patterns, gastrointestinal tract, microflora, nutrition, growth

## Abstract

Free range feeding pattern puts the chicken in a mixture of growth materials and enteric bacteria excreted by nature, while it is typically unique condition materials and enteric bacteria in commercial caged hens production. Thus, the gastrointestinal microflora in two feeding patterns could be various. However, it remains poorly understood how feeding patterns affect development and composition of layer hens’ intestinal microflora. In this study, the effect of feeding patterns on the bacteria community in layer hens’ gut was investigated using free range and caged feeding form. Samples of whole small intestines and cecal digesta were collected from young hens (8-weeks) and mature laying hens (30-weeks). Based on analysis using polymerase chain reaction-denaturing gradient gel electrophoresis and sequencing of bacterial 16S rDNA gene amplicons, the microflora of all intestinal contents were affected by both feeding patterns and age of hens. Firmicutes, Bacteroidetes, Actinobacteria, Proteobacteria, and Fusobacteria were the main components. Additionally, uncultured environmental samples were found too. There were large differences between young hens and adult laying hens, the latter had more Firmicutes and Bacteroidetes, and bacterial community is more abundant in 30-weeks laying hens of all six phyla than 8-weeks young hens of only two phyla. In addition, the differences were also observed between free range and caged hens. Free range hens had richer Actinobacteria, Bacteroidetes, and Proteobacteria. Most of strains found were detected more abundant in small intestines than in cecum. Also the selected Lactic acid bacteria from hens gut were applied in feed and they had beneficial effects on growth performance and jejunal villus growth of young broilers. This study suggested that feeding patterns have an importance effect on the microflora composition of hens, which may impact the host nutritional status and intestinal health.

## Introduction

The intestinal tract is home to 100s of bacterial species, referred to collectively as the intestinal microflora (Ubeda et al., 2017). The significant role of gastrointestinal (GI) microflora in digestion, absorption, health, productivity and other physiological functions has been well recognized (Pourabedin and Zhao, 2015), moreover the intestinal microflora may also protect hosts from pathogens (Waite and Taylor, 2014) and inflammatory bowel diseases to improve gut health (Giacomin et al., 2016); also Lin et al. (2016) studied that intestinal microbiota were necessary for transformation of flavonoids to provide health benefits for the prevention and treatment of some types of chronic diseases. Therefore, it is very relevant to assay the composition of the GI microflora (Barbosa et al., 2016). Information about the composition of the GI microflora in animals is not homogeneous, and affected by different factors. For instance, recent research indicated that the alcohol administration induced shifts in various bacterial phyla in the cecum of mice (Lowe et al., 2017). In general, diet (Stanley et al., 2013; Zhang et al., 2016) and feed additives (Danzeisen et al., 2011) are the most common factors that can impact the GI microflora with respect to diversity, composition, and structure. Jung-schroers et al. (2016) studied that feeding of MacroGard resulted in a more diverse intestinal community that could help to prevent the invasion and establishment of pathogenic micro-organisms. But studies on avian GI microflora have been done more with broilers, the literature about the microflora of laying hens is scarce. Moreover, there are very few studies describing the microflora of chicken and the change between young and adult birds (Han et al., 2016), especially examined the microflora in the GI tract of hens with different age.

Despite considerable available information about poultry, feeding environment is one factor impact the chicken GI microbiota. The intestinal microflora in animals including mammals and chickens all develops in the early stage of life. When young chicks are delivered from the hatchery to a chicken house (typically at the age of 1–2 days), their initial GI microflora is very simple containing a very small number of bacteria belonging to a few species (Hiett et al., 2013). After being placed in different housing system including free range out the house in the natural conditions and commercial patterns in cage, with different materials, chicks are exposed to several sources of bacteria that can gain entry into the immature gut. These exogenous sources of bacteria include feed, water, and ambient conditions (Wang et al., 2016). Because there is little colonization resistance in the young GI tract, many bacteria can readily colonize therein. As young chicks grow, their GI microflora undergoes a series of temporal successions (van der Wielen et al., 2002) and becomes increasingly diverse and complex. Therefore, feeding pattern can have a significant effect on the development process of GI microflora and its eventual composition and structure in chickens.

Several researches have studied the intestinal microflora in poultry including chickens, but only one research assayed the intestinal microflora of chicken affected by feed environmental condition like litters. That study revealed that the fresh litter and reused litter affected the GI microflora of broiler chickens (Wang et al., 2016). While that study pioneered a new area of research, the feeding condition, not just the litter but housing system could affect the GI microflora of chickens. Therefore, our study’s objective was to investigate what influence that feeding patterns would cause on intestinal microflora of hens. The results could be useful for comprehending the correlation between feeding patterns and intestinal microflora of hens as it relates to the growth performance and health condition of chickens by housing system chosen.

## Materials and Methods

### Experimental Animals and Sample Collection

Twenty-five Lindian Chicken with similar weight and same age of 8-weeks or 30-weeks separately were randomly selected from the cage-fed (Daqing Xinghe farm, Daqing, China) and free range groups (Lindian town, Daqing city, China). The samples of small intestinal and cecal digesta of each chicken were collected. The samples from five chickens at same age and same feeding pattern were thoroughly mixed separately by vortex mixing to increase duce homogeneity, and the mixed digesta was divided into new tubes with 2 g mixed sample per tube. One part of the samples was stored at -80°C until further analysis, one part of the samples was diluted by sterile saline to isolate functional *Lactic acid bacteria* strains.

### DNA Extraction and PCR Amplification of the Bacterial 16S rDNA Fragments

The SDS high-salt extraction protocol was performed to extract the genomic DNA from the intestinal contents of the chickens under study (Tapia-Paniagua et al., 2010). The extracted DNA was subsequently purified using the bacterial genomic DNA Extraction Kit (Haibo biological Co., Ltd., Shanghai, China), and stored at -20°C. Bacterial universal primers, 338F: CCT ACG GGA GGC AGC AG and 518R: ATT ACC GCG GCT GCT GG, with the forward primer having a 40 bp GC clamp attached to its 5′ end (CGCCCGGGGCGCGCCCCGGGGCGGGGCGGGGGCGCGGGGGG), were used to amplify the V3 region of bacterial 16S rDNA gene (Yu et al., 2010; Gnanandarajah et al., 2012). The PCR amplification was performed with the followed cycling conditions as previous study descripted (Ramprasad et al., 2007): pre-degeneration at 94°C for 5 min; followed by 30 cycles of 94°C for 1 min (degeneration), 55°C for 45 s, 72°C for 1 min; and a final extension at 72°C for 10 min. Finally, a ∼200 bp DNA fragment was generated for further denaturing gradient gel electrophoresis (DGGE) analysis.

### DGGE Analysis of PCR Products

Ten microliter of the PCR product was analyzed by DGGE (Santos et al., 2008). The denaturing gradient was set at 35–55%, the concentration of the polyacrylamide gel was 8%, and the chemical denaturants used were 7 mol/L of 100% urea and 40% (v/v) acrylamide. Electrophoresis was performed in 1 × TAE buffer, at 150 V, 60°C for 5 h, as previous study descripted (D’Amours et al., 2008).

### Recovery and Sequencing of the Bands from DGGE Gel

Poly-Gel DNA Extraction Kit (OMEGA) was used to recover the DGGE bands. PCR amplification was performed using 338F (CCT ACG GGA GGC AGC AG)/518R (ATT ACC GCG GCT GCT GG) as primers and 2 μl of the amplified product as template. The re-amplified DNA fragments were extracted from the gel, purified, ligated to the pMD18-T vector, and transformed into DH5α competent cells (Anderson et al., 2010). The positive clones were screened and subsequently sequenced at the Beijing Huada Gene Research Center, Beijing, China.

### Data Analysis

Polymerase chain reaction-denaturing gradient gel electrophoresis (PCR-DGGE) fingerprints of the bacteria from chicken intestines were separated using the Quantity One software (Bio-Rad, Hercules, CA, United States), and the homology comparison was performed through a BLAST (Altschul et al., 1997) search in GenBank to obtain 16S rDNA sequences of the most similar and typical isolates.

### Lactic Acid Bacteria Isolation and Culture

*Lactic acid bacteria* from all hens intestinal digesta were isolated by the specific medium. The biological characteristics of the isolated strains were determined by growth test, acids production, antibacterial test, acid and bile salt resistance, intestinal cell adhesion to screen probiotics. The selected isolates were identified by 16S rDNA sequences analysis and were cultured and prepared at 10^6^cfu/g into chicken feed.

### Effects of Mix Lactic Acid Bacteria on Growth and Gut of Broilers

One hundred and twenty AA male broilers aged 1 day were selected and were randomly divided into two groups: control group fed with regular commercial diet, trial group fed with mixed strains feed, five replicates in each group with 12 chicks in each repeat. The experimental period was 28 days, weight and feed intake of chicks were recorded on days 1, 15, and 29. And on the end of the experimental at day 29, five broilers in each group with one chick in one repeat were selected separately to collect the proximal and distal jejunal segments divided into short sections and placed in histology cassettes and stored in the formalin for further histological study by haematoxylin and eosin staining as previous study descripted (Lackeyram et al., 2010). And the digesta in cecum was collected to culture and assay the numbers of Lactic acid bacteria and *Escherichia coli* on culture medium plate. The animal study was approved by the Ethics Committee of Heilongjiang Bayi Agricultural University (Daqing, China) [approval no. SYXK(Hei) 2012-2067].

### Statistical Analysis

Data assay and statistical analysis was performed by Statistical Analysis System version 9.2 (SAS Institute, Inc., Cary, NC, United States). Data of broiler growth performance were expressed as the mean ± standard deviation. *P <* 0.05 was considered to indicate a statistically significant difference.

## Results

### Bacterial Counts

**FIGURE 1:**
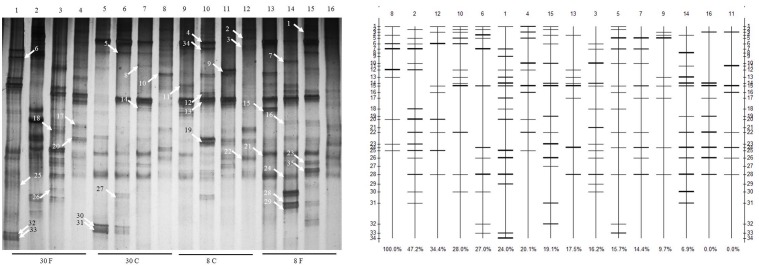
Changes in the structure and degradation capability of stains and cluster analysis of denaturing gradient gel electrophoresis (DGGE) profiles of communities from different hens fed in different patterns with age growing. Duodenum, jejunum, ileum, and cecum samples from: 30-week-old, free range hens (lanes 1–4); 30-week-old, caged hens (lanes 5–8); 8-week-old, caged chickens (lanes 9–12); 8-week-old, free range chickens (lanes 13–16). A total of 35 isolates were detected in the small intestines, and different bands were observed in different intestinal parts of different chickens. Denaturing gradient gel electrophoresis profiles illustrate the structure of the communities. The profiles and bands of DGGE were analyzed using Quantity One software 4.3. The clustering method is by unweighted pair-group method with arithmetic means.

**FIGURE 2:**
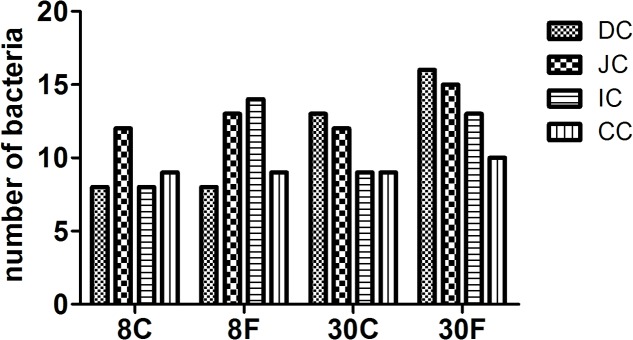
Numbers of bacterial counts in intestinal digesta of hens fed in different patterns. The bar graphic signals were the first character: D for duodenum, J for jejunum, I for ileum, C for cecum, the second character C means contents. 8 and 30 means age of hens, followed C and F presented fed in cage or free range.

The microflora in duodenum, jejunum, ileum, and cecum of 8- and 30-week-old caged and free living chickens were compared in this study. Although DGGE results presented certain common bands among different types of chickens under study, a greater number of bacteria were revealed in the 30-week-old adult chickens and free range chickens as compared to the 8-week-old and caged chickens. Additionally, the total number of bacteria in the duodenum of different chickens showed the biggest difference (**Figure 1**). The bacterial species in chickens with different feeding patterns were various. The 8-week-old cage-fed young hens showed a total bacterial count of 8, 12, 8, and 9 isolates, while the free fed young hens revealed a count of 8, 13, 14, and 9 isolates in the duodenum, jejunum, ileum, and cecum, respectively. Caged 30-weeks laying hens had 13, 12, 9, and 9 bacterial species, while the free range laying hens showed 16, 15, 13, and 10 isolates in these intestinal parts, respectively (**Figure 2**).

### Bacterial Diversity

Based on the proportion of identical bacterial species present among the total bacterial species in the intestinal tract, the microbial diversity was calculated, which are presented in **Table 1**. A major difference in the intestinal bacterial community was observed between the cage and free range chickens, and also between young and adult hens. The shannon-Wiener index and richness of the samples all showed that the intestinal bacterial community in 30-week-old hens fed in free range was significant higher (*P* < 0.05) than three group hens, also 8 weeks young hens were observed the smallest bacterial communities and richness compared with other three group hens (*P* < 0.05). As the same time, compared between different patterns with same age, high evenness and richness were recorded of the hens fed free range.

**Table 1:** Microbial diversity analysis of samples in different patterns (F: free range, C: caged) and ages (W: 30 and 8 weeks).

Treatments	Number of bacterial sequences	Shannon-Wiener index (H)	Evenness (E)	Richness (S)
30W-F	54 ± 5.26^a^	2.55 ± 0.22^a^	0.98 ± 0.01^ab^	13.50 ± 2.65^a^
30W-C	42 ± 3.28^b^	2.28 ± 0.17^b^	0.97 ± 0.01^b^	10.75 ± 2.06^b^
8W-F	44 ± 5.21^b^	2.35 ± 0.26^ab^	0.99 ± 0.005^a^	11.00 ± 2.94^ab^
8W-C	37 ± 2.68^c^	2.15 ± 0.20^b^	0.97 ± 0.01^b^	9.25 ± 1.89^b^


*In the same column, different lowercase letters mean significant difference, *P* < 0.05.*

**FIGURE 3:**
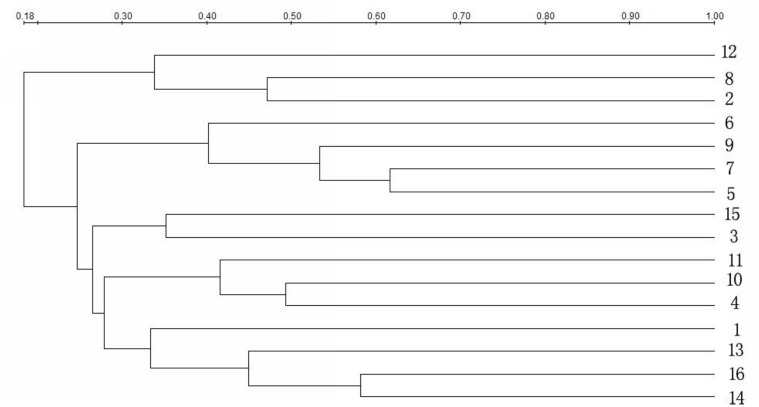
Similarity index of bacterial community in intestines of hens fed in different patterns. Duodenum, jejunum, ileum, and cecum samples from: 30-week-old, free range hens (numbers 1–4); 30-week-old, caged hens (numbers 5–8); 8-week-old, caged chickens (numbers 9–12); 8-week-old, free range chickens (numbers 13–16).

**FIGURE 4:**
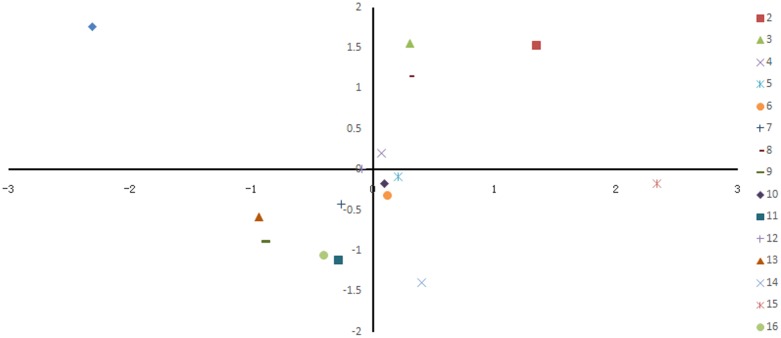
Principal coordinate analysis of 16 samples based on denaturing gradient gel electrophoresis fingerprinting. Points of different colors or shapes represent different samples, the distance among the samples indicated the similarity of bacteria in intestinal different sections of hens.

The strain genetic similarity was calculated and presented in **Figure 3**. The genetic similarity coefficient of isolates from the same intestinal part of the same-age chickens was very low. For 30-week-old chickens, the genetic similarity coefficient of isolates in small intestines of caged hens and free range hens was 18.9–33.5%; while for the 8-week-old chickens, it was revealed to be 17–33.3%. With the same feeding pattern, the genetic similarity coefficient of isolates from the same intestinal part of different-age chickens was slightly higher. The similarity coefficient among intestinal bacterial species in 8- and 30-week-old chickens, for free range raising pattern was 19.4–35.2%, while for the cage-fed chickens, it was 33.1–49.1%; evidently, the values were higher in the caged chickens. PCA analysis was showed in **Figure 4**. For 30-weeks free range laying hens, 1, 2, 3, and 4 as bacterial community in duodenum, jejunum, ileum, and cecum, were decentralized distribution with lower similarity. 5, 6, and 7 as bacterial community in duodenum, jejunum, ileum of 30-weeks caged laying hens, were concentrated distribution with higher similarity, also with high similarity with 10 and 12 as bacterial community in jejunum and cecum of 8-weeks caged young hens, which indicated cage model had more stable effect than free range model on microflora in small intestines. 9 and 13 as bacterial community in duodenum of 8-weeks caged and free range young hens, were concentrated distribution with similarity, none same in laying hens, indicated that feeding environment affected microfloral similarity with age growing.

### Pattern Related Bacteria at Each Section of GI

A total 176 re-amplified DNA fragments following DGGE analysis were presented and analyzed. The phylum and class are shown in **Table 2**.

**Table 2:** Bacterial numbers of all the phyla in the data base of the classes of the most abundant are shown.

Phylum	Class	Bacterial numbers	Diff in patterns	Diff in ages	Diff in GI sections
Firmicutes	*Bacillus*	29 ± 2.15^b^	C	–	S
	*Coprococcus*	35 ± 3.41^a^	F	A	S
	*Clostridium*	28 ± 1.95^b^	F	A	S
Actinobacteria	*Gardnerella*	15 ± 1.32^c^	–	A	S
	*Streptomyces*	11 ± 1.36^d^	F	–	S
Uncultured bacterium	*Uncultured bacterium*	26 ± 2.26^b^	F	–	S
Bacteroidetes	*Bacteroides*	5 ± 0.62^e^	–	–	S
	*Butyricimonas*	5 ± 0.98^e^	F	Y	S
	*Paraprevotella*	8 ± 1.68^d^	F	–	S
	*Alistipes*	3 ± 0.23^f^	–	A	S
Fusobacteria	*Fusobacterium*	9 ± 1.21^d^	–	A	S
Proteobacteria	*Acinetobacter lwoffii*	1 ± 0.32^g^	F	Y	S
	*Pseudoxanthomonas mexicana*	1 ± 0.23^g^	F	A	S


*The “Diff” columns indicate whether abundance was significantly higher in free range (F) or in cage (C), in adults (A) or in young (Y), in small intestine (S) or in typhlon (T), or not significant (–) with a two-tailed *t*-test at *P* < 0.05. In the Bacterial numbers column, different lowercase letters mean significant difference, *P* < 0.05.*

The bacterial species residing in the intestine of hens were treated as five phyla, including Firmicutes, Actinobacteria, Bacteroidetes, Fusobacteria, and Proteobacteria, also some uncultured bacterium, and hens with different age and fed in different pattern shared the same phyla of bacteria: Firmicutes, Actinobacteria, and Fusobacteria excepts Proteobacteria and Bacteroidetes. Thirty weeks laying hens had significantly more sequences of six phyla, while 8-weeks young hens had more abundance of only two.

Total two strains of Proteobacteria were isolated only in small intestines of free range hens, one was from jejunum of 8-weeks free range young hens, another one was from ileum of 30-weeks free range laying hens.

Total nine strains of Fusobacterium, including only one *Faecalibacterium prausnitzii* detected in small intestine of 30-weeks laying hens fed free range and eight strains of *Fusobacterium plautii* found in each samples and three incecum.

The four classes of Bacteroidetes were *Bacteroides*, *Butyricimonas*, *Paraprevotella*, and *Alistipes*, total 21 identifications. Both *Butyricimonas* and *Paraprevotella* were more abundant in free range hens, and *Butyricimonas* was significant more abundant in 8-weeks young hens. Additionally, *Bacteroides*, *Butyricimonas*, and *Alistipes*, were only detected in small intestines, only one strain of *Paraprevotella* was found from cecum. At the same time, only three isolates of *Alistipes* were analyzed and was observed only in the small intestine of 30-weeks laying hens.

Total 26 isolates of Actinomycetes, including *Gardnerella* and *Streptomyces* were detected. Fifteen strains of *Gardnerella* were significant abundant in 30-weeks laying hens, especially in adults hens fed in free range, and only two strains of *Gardnerella* were found in cecum. Total 11 isolates of *Streptomyces* were detected only in the small intestine of hens, and only one strain was found in 8-weeks young hens fed in cage, most were detected in 30-weeks laying hens.

However, there 26 isolates of uncultured bacterium were detected, and were significant abundant in free range hens both of 30-weeks laying hens and 8-weeks young hens, also were more abundant in small intestines.

Out of the 176 isolates of bacteria detected in total, 92 strains belonged to Firmicutes, which were more than 52% of the all isolates. Three classes were detected in Firmicutes, including *Bacillus*, *Coprococcus*, and *Clostridium*. Both *Coprococcus* and *Clostridium* were significant abundant in 30-weeks laying hens fed in free range, while *Bacillus* was more abundant in caged hens and no significant different community in age. All Firmicutes were more abundant in small intestines.

### Lactic Acid Bacteria Isolation and Culture

**FIGURE 5:**
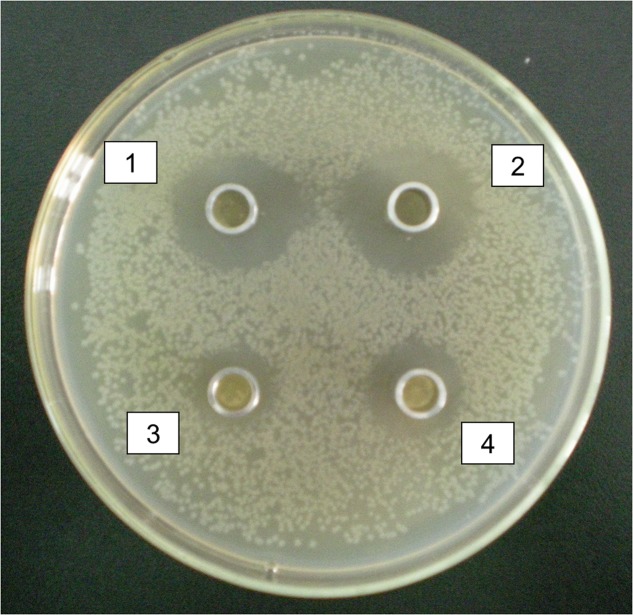
Antibacterial characters of selected isolated *Lactic acid bacteria.* 1 and 2 were antibacterial effect of *Lactococcus raffinolactis* and *Lactobacillus agilis* on *Escherichia coli* with obvious inhibition zone from 1.9 to 2.2 cm. 3 and 4 were antibacterial effect of *Lactobacillus aviaries* and *Lactobacillus acidophilus* on *E. coli* with un-obvious inhibition zone from 0.8 to 1.1 cm.

Two *Lactic acid bacteria* strains were selected with good characters from all the intestinal digesta in hens, *Lactococcus raffinolactis* and *Lactobacillus agilis*. Antibacterial characters of selected isolated *Lactic acid bacteria* showed that the isolated *Lactic acid bacteria* had antibacterial effect on 10^5^CFU/ml *E. coli*, but *Lactococcus raffinolactis* and *Lactobacillus agilis* had obvious inhibition zone from 1.9 to 2.2 cm, however, *Lactobacillus aviaries* and *Lactobacillus acidophilus* had un-obvious inhibition zone from 0.8 to 1.1 cm (**Figure 5**).

The result of bacterial adhesion to the intestinal cells was showed in **Table 3**. Bovine serum albumin was used as blank control. *Lactic acid bacteria* and *E. coli* of 10^5^CFU/ml were adhered to mucin glycoprotein in both duodenum and cecum after cultured for 30 mins, but the adhesion ratio of *Lactic acid bacteria* was significant higher than *E. coli* (*P <* 0.05). Moreover, among the *Lactic acid bacteria*, adhesion of *Lactococcus raffinolactis* and *Lactobacillus agilis* were higher than the adhesion of *Lactobacillus aviaries* and *Lactobacillus acidophilus* (*P* < 0.05).

**Table 3:** Adhesion percentage of the bacteria to the intestinal mucus of broiler (*n* = 3, %).

Strains	Control	Duodenum cell	Cecum cell
*Lactobacillus aviaries*	11.65 ± 1.45^b^	15.24 ± 2.56^b^	17.23 ± 3.15^b^
*Lactobacillus acidophilus*	11.33 ± 1.15^b^	14.03 ± 1.04^b^	16.63 ± 2.06^b^
*Lactobacillus agilis*	11.67 ± 1.58^b^	19.20 ± 1.17^c^	24.46 ± 3.11^c^
*Lactococcus raffinolactis*	11.23 ± 1.36^b^	19.65 ± 2.76^c^	22.45 ± 2.35^c^
*Escherichia coli*	1.24 ± 0.31^a^	3.56 ± 0.47^a^	4.82 ± 0.64^a^


*In the same column, different lowercase letters means significant difference, *P* < 0.05.*

### Effects of Lactic Acid Bacteria Preparation on Growth and Gut of Broilers

The two strains of *Lactococcus raffinolactis* and *Lactobacillus agilis* were mixed cultured in sterilized solid culture medium with bran, skimmed rice bran, rice husk, soybean meal, corn flour, zeolite powder, bentonite and water to make *Lactic acid bacteria* preparation with strains number of 8.7 × 10^9^cfu/g, and prepared with chicken feed at 10^6^cfu/g.

During the whole feeding process, chickens died only in the first week of three chicks in control group and four chicks in preparation group, and the final survival rates of the two groups were more than 93%. The chicks grew well in each group, the results showed in **Table 4**. Compared with the control group, the *Lactic acid bacteria* preparation could increase the weight of chicks significantly in both 2w and 4w (*P <* 0.05). The daily gain of chicks fed with *Lactic acid bacteria* preparation was significantly improved at 3- to 4-weeks by comparing with the control group (*P <* 0.05). The daily feed intake of *Lactic acid bacteria* preparation group was lower than that of the control group (*P* < 0.05) in the whole feeding process, but there was no significant effect of the feed conversion ratio (*P* > 0.05).

**Table 4:** Effect of lactobacillus preparation on growth performance of broilers.

Time	Basic diet	*Lactic acid bacteria* preparation
**Body weight (BW/g)**		
0 weeks	37.17 ± 1.50	37.78 ± 0.40
2 weeks	307.67 ± 10.92	341.58 ± 19.72*
4 weeks	854.65 ± 26.97	935.18 ± 39.53*
**Average daily gain (ADG/g)**		
1–2 weeks	19.32 ± 1.50	21.07 ± 0.40
3–4 weeks	39.07 ± 1.29	42.40 ± 0.98*
1–4 weeks	30.89 ± 0.34	31.56 ± 0.26*
**Daily Feed Intake (DFI/g)**		
1–2 weeks	29.95 ± 1.50	31.39 ± 0.40
3–4 weeks	84.96 ± 1.29	84.79 ± 0.98
1–4 weeks	59.93 ± 1.34	57.76 ± 0.26*
**Feed conversion ratio (FCR)**		
1–2 weeks	1.55 ± 0.12	1.49 ± 0.08
3–4 weeks	2.14 ± 0.55	2.00 ± 0.57
1–4 weeks	1.94 ± 0.24	1.83 ± 0.39


*^∗^Different from the control group in same row, *P* < 0.05.*

The results of the population of *Lactic acid bacteria* and *E. coli* in the cecum of broiler chickens are shown in **Table 5**. Compared with the control group, the number of *Lactic acid bacteria* in the cecum of broiler chickens was significantly higher (*P <* 0.05), as while the amount of *E. coli* was significantly decreased in the broiler fed with *Lactic acid bacteria* preparation (*P <* 0.05).

**Table 5:** Population of lactobacillus and *E. coli* in cecum of broiler (log10 CFU/g)

Bacterial number	Basic diet	*Lactic acid bacteria* preparation
*Lactic acid bacteria*	8.08 ± 0.26	8.51 ± 0.24^∗^
*Escherichia coli*	7.62 ± 0.20	6.38 ± 0.14^∗^


*^∗^Different from the control group in same row, *P* < 0.05.*

*Lactic acid bacteria* preparation also increased (*P* < 0.05) the proximal and the distal jejunal villous height by 14 and 24%, respectively, but it reduced (*P* < 0.05) the crypt depth by 25 and 27%, respectively, in these intestinal segments (**Table 6**).

**Table 6:** Mucosal morphology in the jejunum of broilers fed with Lactic acid bacteria preparation diet and basic diet.

Item	Basic diet	*Lactic acid bacteria* preparation
**Proximal jejunal segment**		
Villus height, μm	646.25 ± 25.90	738.33 ± 22.35*
Crypt depth, μm	294.58 ± 19.44	220.41 ± 17.66*
Villus height:crypt depth	2.20 ± 0.02	3.35 ± 0.04*
**Distal jejunal segment**		
Villus height, μm	652.91 ± 15.30	810.83 ± 38.89*
Crypt depth, μm	349.58 ± 24.12	253.33 ± 11.17*
Villus height:crypt depth	1.87 ± 0.02	3.20 ± 0.06*


*^∗^Different from the control group in same row, *P* < 0.05.*

## Discussion

Since the species of the animal (Li et al., 2012), its rearing environment, and temperature (Lu et al., 2012) greatly influence the intestinal microbiota, their structure and characteristics are not thoroughly reflected through the traditional culture methods. Currently, the largest volume of sequence data exists for intestinal microbial communities, facilitating direct comparisons across multiple studies, which is hindered by some data types such as T-RFLP or DGGE (Oakley et al., 2014). This study for the first time used DGGE technique in examining the feeding patterns impact in the gut microbiota of hens, taking into consideration their different feeding patterns and age. The composition of the GI microflora of hens was known from few studies. Janczyk et al. (2009) fed *Chlorella vulgaris* to laying hens and sequenced bacteria formed ceca were closely related to similar microbiota as the rumen of ruminants like Ruminococcaceae, Lachnospiraceae, and Lactobacilli, giving further insight into still poorly discovered intestinal microbiota of laying hens, especially fed in different patterns. Consistent with the previous study, we also found the same microflora in the cecum in laying hens, and investigated much more bacterial diversity (13 vs. 2 genera) and more detailed differences in microflora community between young and adult hens fed in two patterns of cage and free range. Firmicutes, Actinobacteria, and Bacteroides are the most common phyla in the animal GI including poultry and human, with remainder accounting of Proteobacteria, as the similar results from recent study. Drissi et al. (2015) studied that on the basis of homologies to available bacteria sequences, 175 bacteria were found to been coded in Firmicutes, 79 in Proteobacteria, 34 in Bacteroidetes, and 25 in Actinobacteria. Feng et al. (2017) fed Zn ONPs to hens and found the predominant bacterial community in the ileum belongs to the phylum Firmicutes.

The small intestine harbors large (10^9^–10^11^ cfu/g) bacterial populations dominated by Lactobacillus, Enterococcus, and various Clostridiaceae (van der Wielen et al., 2002; Rehman et al., 2007; Kohl, 2012; Pan and Yu, 2014; Stanley et al., 2014; Waite and Taylor, 2014). Oakley et al. (2014) reviewed to provide additional details regarding the taxonomic composition of microbial communities typically found in the different sections of the GI tract. Although the ceca is looked as organs that digesta resident for 12–20 h for longest time in the intestine and have fermentations contributing to intestinal health and nutrition, especially harbor the highest microbial cell densities in old studies (Lithgow et al., 2014; Sergeant et al., 2014), chickens can survive with experimentally removed ceca. In the present study, the microflora was found clearly separated in different intestinal sections of hens. Several previous studies got the similar results; for example, Sekelja et al. (2012) also reported that a clear separation of microbial composition was seen between ileum and lower gut (cecum and colon) in broiler chickens; Looft et al. (2014) reported that ileum, cecum, and colon have different microbial communities in swine at the phylum and genus level. That these distinctions may be due to different nutrient requirements, is a critical factor for colonizing the commensal bacteria because each section has different nutrient factors (Deusch et al., 2015).

But in our study, reverse results were observed in the small intestines and cecum; only 34 sequences out of total 176 were found in cecum of all hens; more phyla were detected in small intestines, especially Proteobacteria, Actinobacteria, and Bacteroidetes, three phyla were barely none in cecum of both the young hens and laying hens. While recent research used PCR-DGGE analysis revealed that the microbial communities in the cecum of Tibetan Chickens (a local chicken in China) were composed of 16 phyla, moreover Bacteroidetes were represented in cecum at more than 47% (Zhou et al., 2016). These varied microbial community distinction in GI might are caused by different features of each section of GI (Han et al., 2016). For example, ileum has an important role in absorbing nutrition from digested feed, which might influence the microflora in ileum; there is an anaerobic condition in cecum, which makes cecum the fermentation pot for some bacteria dislike oxygen. Also some research investigated that intestinal microflora disorders or imbalance might be relevant to some intestinal diseases like inflammatory bowel disease or obesity-associated diabetes (Chang et al., 2008; Michail et al., 2012; Murri et al., 2013; Huang et al., 2017). On the contrast, Mejia-Leon et al. (2014) indicated that microflora community was not corresponded with these diseases. According to these converse results, further studies may need to conduct.

When investigating the intestinal microflora affected by different environmental factors, some materials from conventional laboratory animals were used as control comparisons. However, what is normal as control? Norin (2011) concluded from several studies that the control materials have to derive from normal/healthy materials in either human or animal. In our study, both free range hens and caged hens were totally healthy and no abnormal hens were used for the performed assay of intestinal microbiota, so no other kinds of control material were used in the study. Our results showed that the microflora of free range hens and laying hens was 21served to be more diverse as compared to the caged hens and young hens, respectively; and the results investigated that the backyard environment was more unpredictable, and the free range hens stored a greater variability of bacterial species. In contrast, the captive environment was reported to be relatively stable. Hens raised in cages revealed a higher similarity coefficient among the intestinal bacterial species as compared to the hens reared in backyard. And as descript in previous study, compared with the complex and resilient adult microbiome, intestinal microflora in young animal including children and young chicks is intrinsically plastic, deeply affected by few variables but less exposed to factors that may change its composition (Buccigrossi et al., 2013). The present study got the similar results that adult hens composed more and complex strains in their intestine than the young chicks.

In the most bacteria isolated from gut digesta, *Lactic acid bacteria* strains have generally recognized as safe (GRAS) status, gaining popularity for application in dairy products and play probiotic roles in human and animal’s GI tract (Giraffa, 2014; Bian et al., 2016a). And some kinds of *Lactococcus* and *Lactobacillus* have shown strong capacities against artificial gastric juice, intestinal juice, and bile salt (Bian et al., 2016a). Even, some *Lactobacillus* could effectively alleviate diarrhea in mice via modulation of intestinal microflora and improve the function of immune system (Bian et al., 2016b), and could enhance the population of beneficial bacteria and reduced the population of Enterobacteriaceae (Thakur et al., 2016). In this study, *Lactococcus raffinolactis* and *Lactobacillus agilis* were selected from intestinal digesta of hens with acid and bile salt tolerance, and higher adhesion to intestinal cell, they both showed growth improvement on broiler chicks, and improve the nutrition absorption of gut by affecting the jejunal villous growth. As while, *Lactococcus raffinolactis* and *Lactobacillus agilis* had antibacterial activity on *E. coli* both *in vitro* and *in vivo* to maintained intestinal health. There are several researches have proved that *Lactic acid bacteria* isolated from animal gut had beneficial aspects on intestinal tract through their antimicrobial activities. For example, an *Enterococcus faecalis* strainis capable of clearing vancomycin-resistant Enterococcus (VRE) from the intestinal tract of mice through the expression of a plasmid-encoded bacteriocin (Kommineni et al., 2015). As same time, *Lactic acid bacteria* isolated from animal gut must compete for the same nutrient sources with the intestinal pathogen commensal bacterial species and are highly adapted to the gut environment with high tolerance to the intestinal juice and very efficient at obtaining energy from the diet or utilizing host-derived nutrients (Ubeda et al., 2017). We could thus infer that microflora in hens gut especially *Lactic acid bacteria* including *Lactococcus raffinolactis* and *Lactobacillus agilis* had beneficial in the regulation of intestinal health and balance of intestinal microbiological community in young chicks. As concluded previously that targeting intestinal microflora maybe a novel strategy for therapy and prevention of chronic diseases or contributes to nutrient digestion and energy harvest (Buccigrossi et al., 2013).

## Conclusion

Collectively, *Coprococcus*, *Clostridium*, *Gardnerella*, *Alistipes*, *Fusobacterium*, and *Pseudoxanthomonas* bacteria tended to increase with age. *Coprococcus*, *Clostridium*, *Butyricimonas*, *Paraprevotella*, and *Acinetobacter* were more abundant in free range hens. Feeding patterns regimens also had greater impact on gut bacterial community in laying hens than young hens. Small intestinal bacterial community was affected more profoundly than cecal digestal bacterial community. These findings indicated that the diversity of gut microbiota was highly influenced by section of the intestine, raising environment, and also affected by feeding patterns and age of chicken. The present study suggested that feeding patterns have an importance effect on the microflora composition of hens, which may impact the host nutritional status and intestinal health (Wang et al., 2016). Also feed supplementation with *Lactic acid bacteria* isolated from intestinal digesta of hens may promote growth performance and good for jejunum villus growth. These changes could be concluded that microflora in hens intestine may provide health benefits to intestinal development by nutrient effects (Wang et al., 2017) at the starter phase.

## Author Contributions

YC and SL conceived the work. QW performed all experiments, analyzed the results, and wrote the manuscript. RS, YZ, and YL assisted in bacterial testing. All authors read and approved the final paper.

## Conflict of Interest Statement

The authors declare that the research was conducted in the absence of any commercial or financial relationships that could be construed as a potential conflict of interest.
